# Supplementary material for: SASP-Dependent Interactions between Senescent Cells and Platelets Modulate Migration and Invasion of Cancer Cells
Source: Int J Mol Sci. 2019 Oct 24;20(21):5292. doi: 10.3390/ijms20215292 (PMC6862446; doi:10.3390/ijms20215292)
Supplement: Supplementary file 1 [file ijms-20-05292-s001.pdf]

Supplementary Materials:

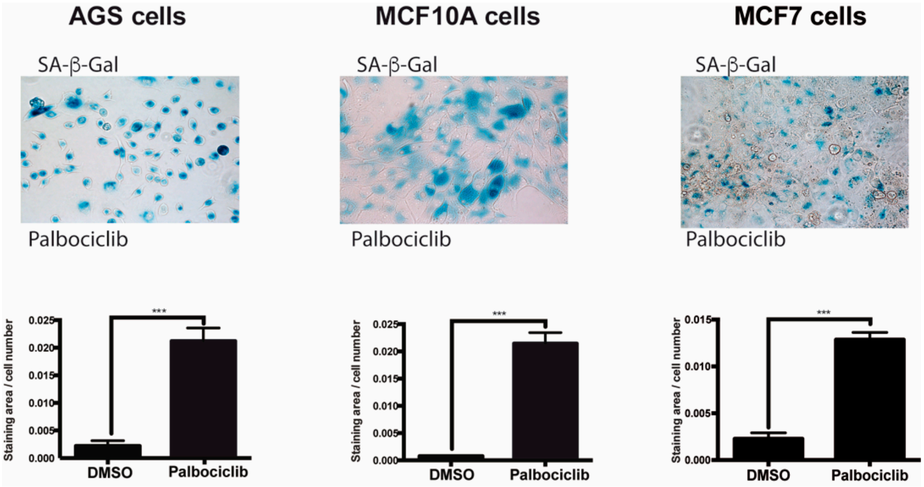

Figure S1. Pharmacological induction of senescence using Palbociclib. Cells were treated for 96 hours with 1 μM Palbociclib and senescence induction was determined by senescent-associated β-galactosidase (SA-β-Gal) assays. The relative proportion of SA-β-Gal positive cells in at least 5 separate microscopic fields was determined by analyzing the colored area in Palbociclib- and DMSO-treated cells using ImageJ program. ($n = 6$; *** $p < 0.001$; error bars, mean ± SD).

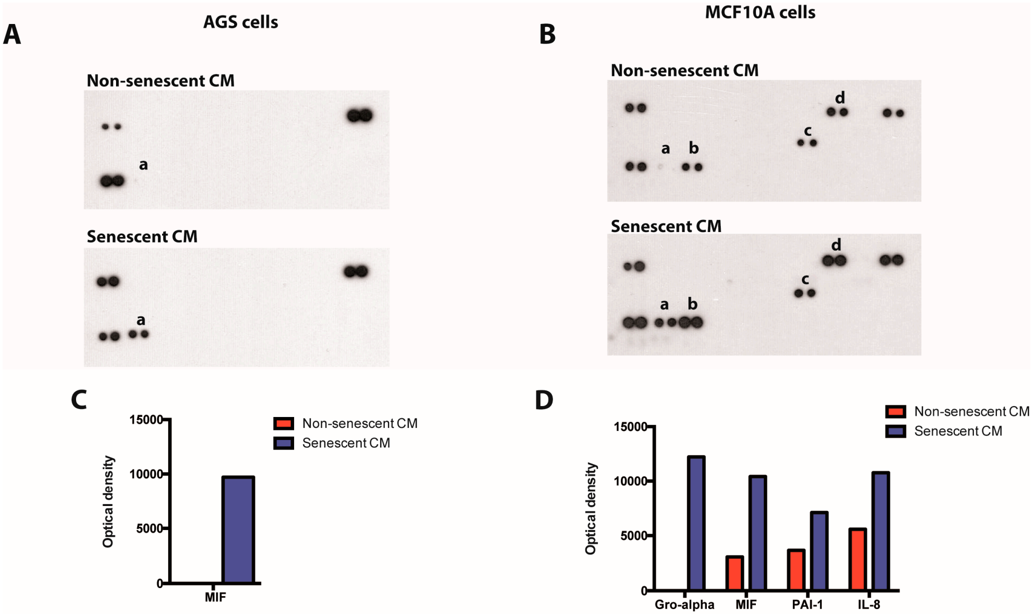

Figure S2. Human cytokine array analyses of media conditioned by senescent and non-senescent cells. After 96 hours of senescence induction, media conditioned by senescent (Palbociclib-treated) and non-senescent (DMSO-treated) cells were subjected to a human cytokine array panel capable of detecting 36 cytokines (panels **A** and **B**). Quantitative densitometric analyses, examining the ratio

between the intensities of signals of the indicated cytokines in conditioned media from Palbociclib- and DMSO-treated cells, were quantified in **C** and **D**. Cytokines that were upregulated in media conditioned by senescent MCF-10A cells compared with media conditioned by DMSO-treated control cells included MIF (Macrophage Migration Inhibitory Factor), PAI-1 (Plasminogen Activator Inhibitor 1), IL-8 and Gro-α (Growth Regulator Protein α). In contrast, only MIF was detected in conditioned media derived from senescent AGS cells (panels C and D). (**a**) MIF; (**b**) Gro-α; (**c**) Serpin-E1; (**d**) IL-8.

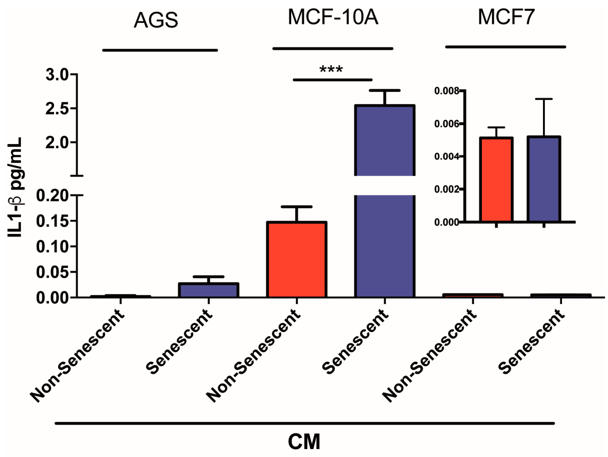

Figure S3. ELISA quantification of IL-1β (in pg/ml) present in media conditioned by senescent and non-senescent AGS, MCF-10A and MCF-7 cells (n = 3; *** $p < 0.001$; error bars, mean ± SD).

Table S1. Primer sets used for quantitative real time PCR analyses.

Gene	GenBank Accession	Forward 5'-3'	Reverse 5'-3'
IL-1 α	NM_000575.4	AGA TGC CTG AGA TAC CCA AAA CC	CCA AGC ACA CCC AGT AGT CT
IL-1β	NM_000576.2	ATG ATG GCT TAT TAC AGT GGC AA	GTC GGA GAT TCG TAG CTG GA
IL-11	NM_000641.3	TTG TCC GAG ATG TCA TGG GTT	TCC TGT CGC TGA TAT TCT CTC C
PAI-1 (serpine1)	NM_000602.4	TAG ACC GAT TAT TGA CCG ACCT	GTT TGC CAC GAG AAT CAA ATC C
MMP7	NM_002423.4	ATG TGG AGT GCC AGA TGT TGC	AGC AGT TCC CCA TAC AAC TTT C
MMP14	NM_004995.3	CAT CTG TGA CGG GAA CTT TGA	GGC AGT GTT GAT GGA CGC A
MMP3	NM_002422.4	ATG TTC GTT TTC TCC TGC CTG TGC	CGA GTG CTT CCC CTT CTC TTG G
MMP1	NM_001145938.1	TCT GGG GAA AAC CTT TCG ACT	CAC CAA CGT ATT CAA AAG CAC AA
MMP9	NM_004994.2	GAC AAG AAG TGG GGC TTC TG	GCC ATT CAC GTC GTC CTT AT
MMP10	NM_002425.2	TGC TCT GCC TAT CCT CTG AGT	TCA CAT CCT TTT CGA GGT TGT AG
GM-CSF	NM_000758.3	TTC TGC TTG TCA TCC CCT TT	TGC CTG TAT CAG GGT CAG TG
G-CSF	NM_000759.3	CAA GCC CTC CCC ATC CCA TGT AT	GGG ATG GGA GGA CAG GAG CTT TT
FN1(Fibronectin)	NM_001278438.1	GAG TTG TCG TGG TCC CTC AG	TGG AGG CGG CAT CAT AGT TG
ICAM-1	NM_000201.2	TCC CTT CCC CCC AAA ACT GAC A	GCT CCC AGT GAA ATG CAA ACA GG
THPO	NM_000460.4	GGT TCA CCC TTT GCC TAC ACC	CCT CCA TCT GGG TTT TCC ATT C
PAI-2	NM_001143818.1	AAA TGG GCT TTA TCC TTT CCG T	AGC TTT TCA CGC AAG TAC ATC A
RL19	NM_000981	CAT CCG CAA GCC TGT GAC	TGT GAC CTT CTC TGG CAT TCG
